# The World’s Northernmost Harbour Seal Population–How Many Are There?

**DOI:** 10.1371/journal.pone.0067576

**Published:** 2013-07-03

**Authors:** Benjamin Merkel, Christian Lydersen, Nigel G. Yoccoz, Kit M. Kovacs

**Affiliations:** 1 Norwegian Polar Institute, Fram Centre, Tromsø, Norway; 2 Department of Arctic and Marine Biology, University of Tromsø, Tromsø, Norway; University of Kent, United Kingdom

## Abstract

This study presents the first abundance estimate for the world’s northernmost harbour seal (*Phoca vitulina*) population, which resides in Svalbard, Norway, based on three digital stereoscopic photographic surveys conducted in 2009 and 2010. The counts from these high resolution 3D images were combined with a novel method for estimating correction factors for animals that were in the water at the time of the surveys, in which extensive behavioural data from radio-tagged harbour seals were used together with age distribution data to estimate the proportion of seals of various age and sex classes hauled out at the times of the surveys. To detect possible seasonal shifts in age distribution between surveys, lengths of hauled out seals were measured from the stereoscopic images. No body-length differences were detected between the surveys; but, this may be due to a high degree of sexual dimorphism exhibited in this population. Applying the modelled correction factors, a total of 1888 (95% CI: 1660–3023), 1742 (1381–3549) and 1812 (1656–4418) harbour seals were estimated for the surveys flown on 01 August 2009, 01 August 2010 and 19 August 2010, respectively. The similarity between the three survey estimates (despite significant differences in the number of animals actually counted on the photos from each survey effort) suggests that the variation in numbers of hauled out seals is reasonably accurately adjusted for by the haul-out probability model. The low population size, the limited spatial distribution of the population and its reduced genetic diversity make this population vulnerable to chance events, such as disease epidemics.

## Introduction

Harbour seals have a broad geographic distribution in coastal waters in the northern hemisphere. The species is categorized into five subspecies, with *Phoca vitulina vitulina* occupying the eastern Atlantic from Brittany to the Barents Sea, including the world’s northern most population located in the high arctic archipelago of Svalbard (78.2°N, 15.5°E), Norway [1]. Harbour seals were first reported to occur in this island group in 1898 [2], but were not the subject of scientific study until the 1970s (reviewed in [3]). The harbour seals in Svalbard constitute a highly genetically distinct population that has limited gene flow and low genetic diversity; this population also displays evidence of having experienced a recent bottleneck [4].

The Svalbard harbour seal population exhibits a high degree of sexual dimorphism compared to more southerly populations with adult males being significantly heavier and longer than adult females [5]. There is also a marked absence of older individuals in this population; the oldest seal registered in an extensive capture programme in the late 1990s was only 22 years old. This lack of older animals is unusual compared to other populations of this species, which have a much higher proportion of individuals in the 15+ yr age categories [6], [7], [8], [9], [10]. Svalbard harbour seals are on the Norwegian Red List and are protected from hunting. Some crude attempts to enumerate this population have been conducted [1], [3], based on counts made from land or sea, but no abundance estimate is available.

The most common method for estimating abundance of harbour seals is to count the number of animals ashore during the pupping or moulting periods. Hauled out seals are often counted either directly (e.g. [11], [12], [13], [14], [15]) or on photographs from aerial surveys (e.g. [16], [17], [18], [19], [20]). The pupping period for harbour seals in Svalbard takes place during the second half of June [21]. Harbour seals usually give birth to a single pup with an adult-like pelt; although a small proportion of pups of this species are born bearing their lanugo coats [22]. Pups are nursed for about 24 days [23] and subsequently weaned around mid July. Harbour seals are able to swim and dive from the day they are born and gradually increase the time they spend in the water with age [24], [25]. Towards the end of the nursing period mature females enter breeding condition and mating occurs, which takes place in the water [26], [27], [28]. Moulting follows the breeding period, taking place from mid July to mid September. The moulting process in individuals takes three to five weeks to complete [29]. Immature seals moult first, followed by adult females and lastly adult males [29], [30], [31].

The pupping and moulting periods constitute the time of the year when the highest proportion of harbour seals is hauled out on shore and thus represents the best times to conduct population surveys. However, even during the peak moulting period there is always a proportion of the population at sea and therefore not visible for counting. Several studies have shown that this proportion varies with temporal and environmental conditions such as; season, time of the day, tidal cycle and various meteorological factors (e.g. [29], [31], [32], [33], [34]). Because of differences in the timing of moult according to sex and age, the proportion of the population counted might not be representative of the sex and age structure of the total population [35]. This natural variation in number of seals hauled out, as well as the age and sex composition of the hauled-out proportions of the population must be taken into consideration during population assessments.

Harbour seals have a restricted distribution within the Svalbard Archipelago. The main haul-out area for this population during pupping and moulting is the west coast of Prins Karls Forland [1]. Pupping has in fact only been observed along this 86 km long coast line [3]. Additionally, a satellite tagging study conducted 1992–1994 [36] concluded that the majority of Svalbard’s harbour seals appeared to be quite stationary around Prins Karls Forland throughout the year.

The purpose of the present study was to provide the first population estimate for harbour seals occupying Prins Karls Forland, using a series of aerial surveys (counting seals on digital stereoscopic photographs), in combination with correction factors based on both behavioural data (haul-out information from VHF tracking) and environmental data in combination with information on population age distribution (based on catch data).

## Methods

### Ethics Statement

All research activities conducted during, and in support of, this study were carried out under permits from the Norwegian Animal Care Authority and the Governor of Svalbard (2009/00103-2 a.512) and followed best practice for all animal handling (Gales NJ, Bowen WD, Johnston DW, Kovacs KM, Littnan CL, et al. (2009) Guidelines for the treatment of marine mammals in field research. Mar Mamm Sci 25∶725-736 [37]).

### Aerial Surveys

**Figure 1:**
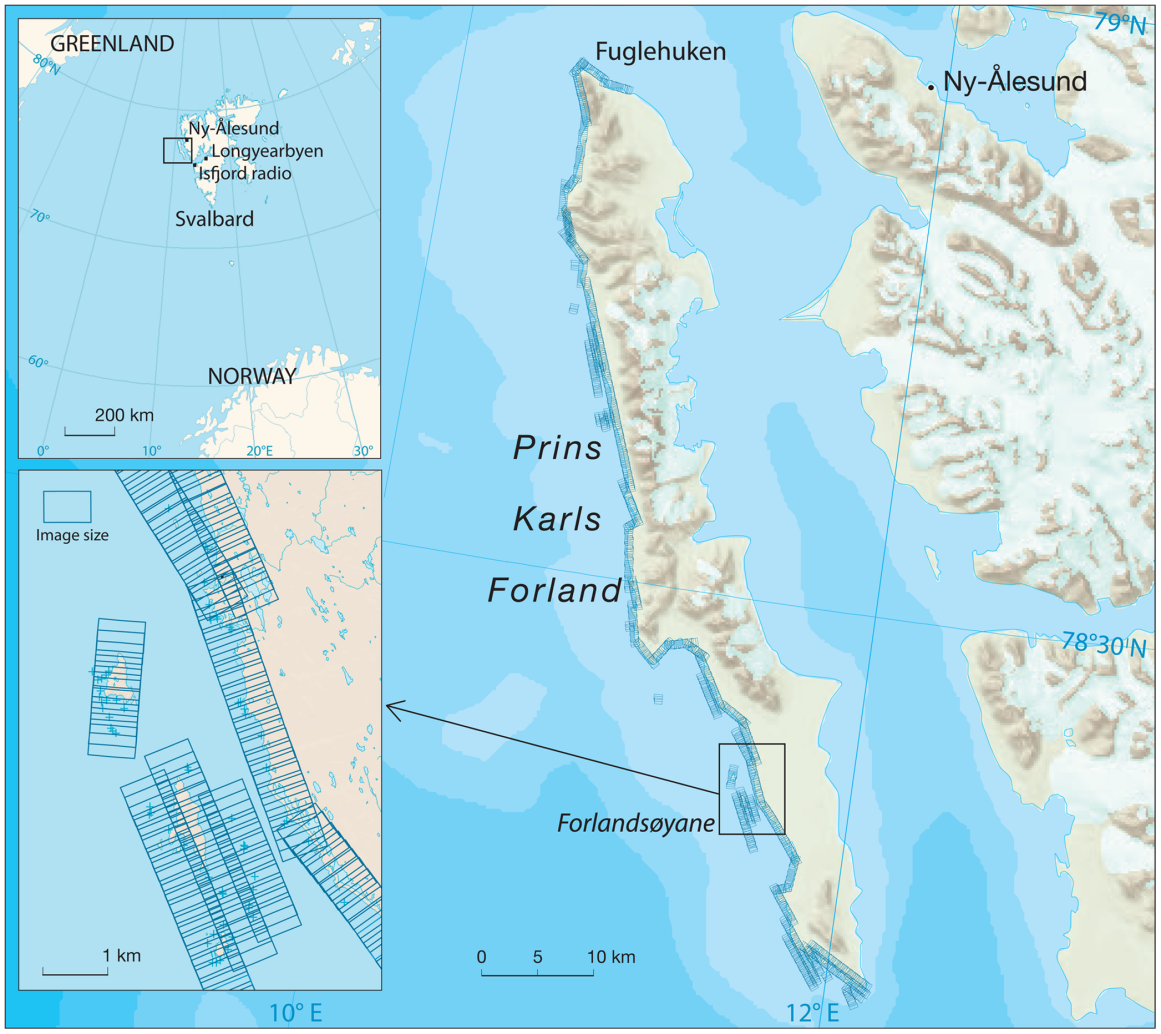
Study location and aerial survey structure. Map of Prins Karls Forland showing the extent of the aerial surveys flown and the locations Fuglehuken and Forlandsøyane. Top left panel shows the locations of Prins Karls Forland, Ny-Ålesund, Longyearbyen and Isfjord Radio within the Svalbard Archipelago. Bottom left panel shows Forlandsøyane and the adjacent coast as an example of the size and structure of the overlapping images taken along the whole west coast of Prins Karls Forland during each of the surveys. Printed with permission from the original copyright holder, Norwegian Polar Institute.

Aerial digital photographic surveys were flown for harbour seals, covering the entire west coast of Prins Karls Forland (Figure 1), on 01 August 2009 and 01 and 19 August 2010 (N = 3). All of the surveys were flown during afternoon low-tide periods, under similar meteorological conditions (sunny, no wind, no clouds). Since these are optimal haul-out conditions for this species [12], [31], [33], [34], it is expected that a maximal fraction of the population was hauled out at the time of the surveys.

A Twin Commander 690C fixed-wing aircraft equipped with a Microsoft Vexcel Ultracam XP (Focal length: 100.5 mm; gyro mount with 5% correction in pitch/roll and 20% correction in yaw; software for image processing: OPC and ULTRAMAP (VEXCEL, Boulder, Colorado, USA)), flew the surveys at an altitude of 670 m. Potential errors in the optics in relation to the film frames are of the order X = 0.120 mm ±0.002 mm and Y = 0.180 mm ±0.002 mm. These sources of error are taken into account in the software calibration for focal length, principal point of symmetry, principal point of autocollimation and radial distortion, such that any remaining error will be less than the size of a pixel in the digital images. The flight plan was designed to cover the whole west coast of Prins Karls Forland including adjacent small islands and skerries (Figure 1). An image was shot approximately every two seconds while flying at 300 km/hr. Each image covered 0.31 km^2^, so that an overlap of 60% between each image was achieved to make stereoscopic visualization possible. In total 2,950 digital stereoscopic images, with a ground sample distance (i.e. pixel size) of 4×4 cm, were manually inspected for the presence of harbour seals using Z/I Imaging Quick View 4.2.0.1 software (Z/I Imaging GmbH, Aalen, Germany). The 01 August 2009 survey (975 images) was double-blind counted in 2D, as well as in stereo, by two readers to assess variation among readers. Seals were relatively easily detected on the digital images and it was possible to distinguish harbour seals from other pinniped species on the images *i.e.* bearded seals (*Erignathus barbatus*) without difficulty (mainly by size). Variation in the number of harbour seals detected by the two different readers was deemed small enough to be ignored (max. 3.3%) and therefore no attempts were made to correct for reader effect. The small difference that did occur between readers was mainly due to uncertainty in counts in a few cases where seals were disturbed and had rushed into the water at the time of the survey. Thus, for the estimations of population size it was assumed that all seals hauled out at the time of the surveys were detected and correctly identified to species.

To explore whether age structure of hauled out seals shifted through the moulting period, two surveys, 2.5 wks apart were flown in the second year of the study. The stereoscopic images enabled measurements of all non-moving seals in the images, including corrections of these measurements to adjust for angles of the substrate the seals hauled out on using SOCET SET 5.5 software (BAE Systems, California, U.S.A.). This made it possible to assess a potential shift in age structure through the moulting season via exploring the length distribution of the hauled out animals. Quantile-quantile plots were used to compare the length distributions of the seals between the different surveys.

### Behaviour Data

In order to be able to create an estimate of the harbour seal population at Prins Karls Forland, the number of seals hauled out during the surveys had to be divided by the estimated proportion of the various age and sex classes hauled out at the time of the surveys, to account for the proportions of animals in different age and sex categories that were in the water. A detailed data set on haul-out behaviour of Svalbard harbour seals was used to facilitate these calculations. Raw data for this estimate were collected during an earlier study on haul-out behaviour of this population [31]. In this earlier study the behaviour patterns of 37 harbour seals equipped with VHF tags (Followit AB, Lindesberg, Sweden) during the pupping and moulting season in 2000 (June to August) were monitored around the clock *via* three automatic receiver stations distributed along the western shoreline of Prins Karls Forland. The raw data from [31] was used to construct a model to predict the probability of seals to haul-out on any given day, time of day, time within tide cycle and temperature. Due to the marked differences in haul-out behaviour of adult females, adult males, immature individuals and pups of the year [31], correction factors were estimated for each of these four groups. The VHF data was filtered by pulse rate using only pulses with ∼1.5 sec intervals for all groups except adult females, where intervals of ∼1.1 sec were retained in the dataset (similar to [38]). Signals were pooled into one hour bins since the 15 min resolution of the receiver scanning of the tags led to computational problems during the model selection process. The time frame for the behavioural component of the study was 07 July to 25 August, because 07 July was the first day when there was no human disturbance (e.g. seal capture work) taking place in the study area and 25 August was the last day a signal was recorded. Records for individual animals went from 07 July to the last day upon which a signal was received for each of the individuals respectively (see Figure S1 in Appendix S1). Six seals were excluded from the analyses because no signals were recorded for them. Consequently, haul-out data from 31 individuals (seven adult females, six adult males, eight juveniles and ten pups) were used as the basis to model a correction factor in this study. The haul-out behaviour of these seals is assumed to be representative for the population as a whole. Tidal data was retrieved for Ny-Ålesund (Figure 1) from the Norwegian mapping authority (http://www.vannstand.no). Temperature data for 2000 were taken from the weather station at Isfjord Radio (Figure 1) (http://www.eklima.no). Only ambient temperature values for the 01 August 2010 aerial survey were available. Therefore, the average of the temperature values from two weather stations in Longyearbyen and Ny-Ålesund (Figure 1) were used for the other surveys. This seems to be quite accurate as the calculated average temperature value for the 01 August 2010 survey only differed by only 0.5°C from the observed value. For statistical analyses and model computations R software version 2.15.1 (R Development Core Team, 2012) was used.

### Data Analyses

A generalized additive mixed effect model (GAMM) using the “mgcv” package (version: 1.7–18) [39], [40] was computed to estimate the probability of haul-out for each of the four age/sex groups. Due to the binary nature of the response variable (presence-absence) a binomial distribution was assumed and a logit-link was utilized. Further, a first-order autoregressive correlation structure (AR1) at the level of the data [41], together with a random inter-individual variance component, was incorporated to account for the temporal correlation between observed values for each tagged seal and the individual differences in haul-out behaviour between tagged seals. This is similar to the approach employed by [42] and [43], but the use of AR1 at the level of the data has been shown to be more flexible and robust [41]. A gamma value of 1.4, to avoid over-fitting, as recommended in [39], and restricted maximum likelihood estimation (REML) were used to estimate parameters of the models (see R script in Appendix S2 and test data in Appendix S3). GAMM models were used because the relationship between season (day of the year, DOY) and the probability of haul-out was strongly non-linear. Thus, this relationship could not be presented adequately using simpler models such as GLMM (generalized linear mixed effect modelling) or low-degree polynomials. Data for each covariate was standardised by subtracting the mean and then dividing by the standard deviation to unify the scale of all variables in order to prevent computation problems, as recommended in [44]. Exploratory gam plots were used to assess the relationship between the response and each covariate. No strong co-linearity between predictor variables was found. DOY, time to and from the nearest low tide (LOW TIDE), time of the day (TIME) and air temperature in °C (TEMP) were examined in candidate models because of their documented effects on harbour seal haul-out behaviour [29], [31], [32], [33], [34]. All were included as continuous variables that were unique to each of the four age/sex groups (GROUP). Cubic spline regression smoothing functions were applied to DOY and LOW TIDE, with initial values for k of k = 5 and k = 4, respectively. A cyclic cubic spline regression smoothing function with an initial value k = 5 was also applied to TIME to ensure circularity of the covariate [39]. TEMP was included as a linear effect. The uncertainty of the mean haul-out probability values (95% confidence intervals (CI)) was calculated as *±2 SE* (standard error) on a logit scale, and the uncertainty in the predictions for a random individual seal was then estimated including both uncertainty in the mean and the random between seal variance (*δ^2^), i.e. ±2(SE^2^+δ^2^)^0.5^*.

Different approaches for model selection were considered: AIC; backwards selection based on P-values; and a priori selection based on knowledge of factors affecting haul-out behaviour [45]. The use of AIC led to either highly complex models (when autocorrelation was not included) or a constant model (when autocorrelation was included), even if in the latter case other models included terms that were highly statistically significant (see Results). Therefore, model selection for fixed effects terms was based on backwards selection, removing terms sequentially using P-value >0.05 as a criterion [39], [46]. This led to models that were biologically well-grounded.

The average TIME, LOW TIDE and TEMP values for the three hours in which each aerial survey took place were used to predict the probability that seals within each of the four age/sex groups would be hauled-out at a given time. An estimated age distribution, based on catch data from a previous study at the same location [5], was used to combine the four estimates. Because juveniles were underrepresented in the data set [5], a linear model on a log scale was computed, based on an assumed 50% pup mortality. A sex ratio for adult seals of 1∶1 was assumed; sex ratios for adult seals of 0.77∶1 to 1∶0.77 were also explored to assess the sensitivity of the 1∶1 assumption. Data from age classes six to 22 years were included directly into the model. The probabilities to haul-out (*p*) for each age/sex group (*j*) for each survey at time (*t*) were multiplied with the proportional representation of each group in the population (*q*) in order to derive an average population correction factor. The total population estimate (*N*) based on the aerial counts (*Y*) for each survey was then calculated using:
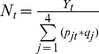



In order to assess the uncertainty in the population estimate due to the estimated haul-out probabilities, a non-parametric bootstrapping approach was used. Individual seals for each age/sex group were sampled with replacement 500 times (see Appendix S2). This gave a series of bootstrapped estimates, which combined with the observed counts at each time point, resulted in a set of population estimates. The 95% quantiles of these estimates were used to derive upper and lower confidence bounds for the predicted population estimates. But, it should be noted that the variance estimated by the bootstrap is conditional on the estimated population structure.

Model validation was achieved by averaging residuals for each individual seal and each covariate and exploring violation of model assumptions (i.e. homogeneity of variance, non-linear relationships). Assumptions appeared to be met.

## Results

A total of 2,950 digital stereoscopic images were inspected for seals both in 2D and in stereo. In total 981, 730 and 1295 harbour seals were counted on the images from the 01 August 2009, 01 August 2010 and 19 August 2010 surveys, respectively (Table 1). The counts obtained using stereo imagery were higher (except for the 01 August 2010 survey), had fewer misidentifications and were more similar between the two readers, compared to counts attained from 2D images (Table 1).

**Table 1:** Date (DOY, Day Of the Year), flight time (averaged for TIME), hours to/from nearest low tide (LOW TIDE), air temperature in °C (TEMP), number of seals counted on the digital aerial survey images in 2D and in stereo as well as the number of disturbed seals on images (for the three aerial surveys).

Survey No.	Date (DOY)	Time of day (TIME)	Hours to/from nearestlow tide (LOW TIDE)	Air temperature (°C) (TEMP)	No. of seals countedon images in 2D	No. of seals countedon images in stereo (*Y*)	No. of disturbed sealson images in stereo
1	01.08.2009	15∶36–18∶06	−0.48	10	897	981	79
2	01.08.2010	11∶14–13∶43	0.32	7	782	730	252
3	19.08.2010	15∶07–17∶29	0.28	9	1245	1295	85

LOW TIDE and TEMP are averaged values for each survey interval.

**Figure 2:**
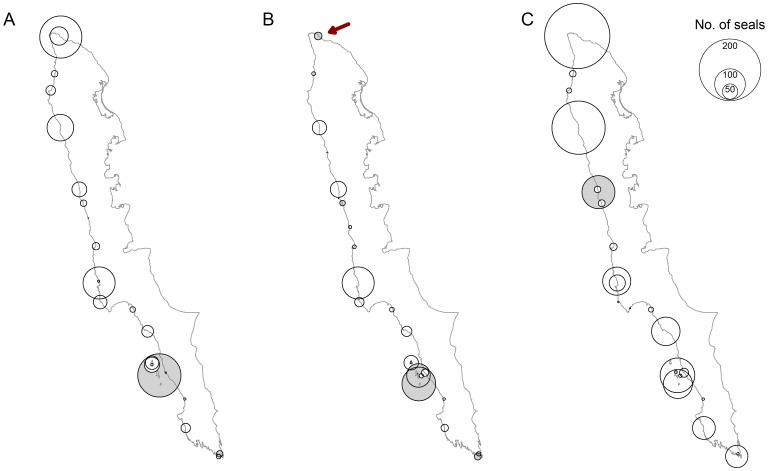
Spatial distribution of hauled out seals. Distribution and group sizes of hauled out harbour seals along the west coast of Prins Karls Forland during three aerial surveys. Shaded circles indicate haul out areas that were disturbed i.e. some animals were moving towards the water. The red arrow indicates a hauled out site that was disturbed by a polar bear. A, B and C represent the survey results from 01 August 2009, 01 August 2010 and 19 August 2010, respectively.

More seals were detected in the 19 August 2010 survey, which was later in the moult, compared to those flown 01 August in either of the two study years. Haul-out groups in the northern part of Prins Karls Forland, as well as along the southern tip of the island, were larger later in the season (Figure 2).

A small fraction of seals were disturbed in each survey effort. Generally these movements toward the water were observed at the same geographical areas in each survey (Figure 2). One identified disturbance factor was the presence of a polar bear (*Ursus maritimus*) near the northernmost haul-out group, at Fuglehuken, during the 01 August 2010 survey (red arrow in Figure 2). The fraction of disturbed seals was highest in this survey (Table 1).

**Figure 3:**
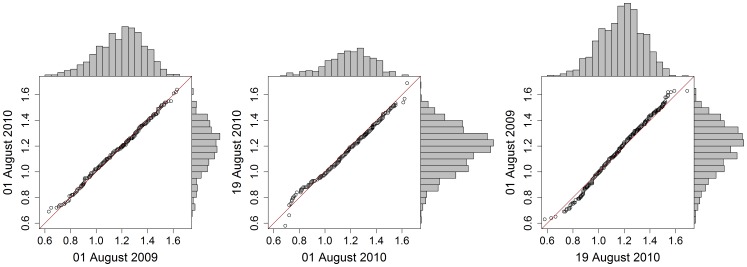
Comparison of length distributions. Quantile-Quantile plots accompanied by seal length frequency distributions, comparing each of the three aerial surveys against each other. The red line in each panel shows the relationship that would reflect complete equality of the two distributions being compared.

The average measured lengths of harbour seals hauled out during the 01 August 2009, 01 August 2010 and 19 August 2010 surveys were 1.19 m (standard deviation (SD) - 0.18 m, range - 0.63–1.63 m, N - 903, 92% of total seals counted), 1.20 m (SD - 0.18 m, range - 0.69–1.64 m, N - 477, 65% of total seals counted), and 1.18 m (SD - 0.17 m, range - 0.58–1.69 m, N - 1187, 92% of total seals counted), respectively. No temporal trend in length distribution could be detected between the three surveys (Figure 3).

**Figure 4:**
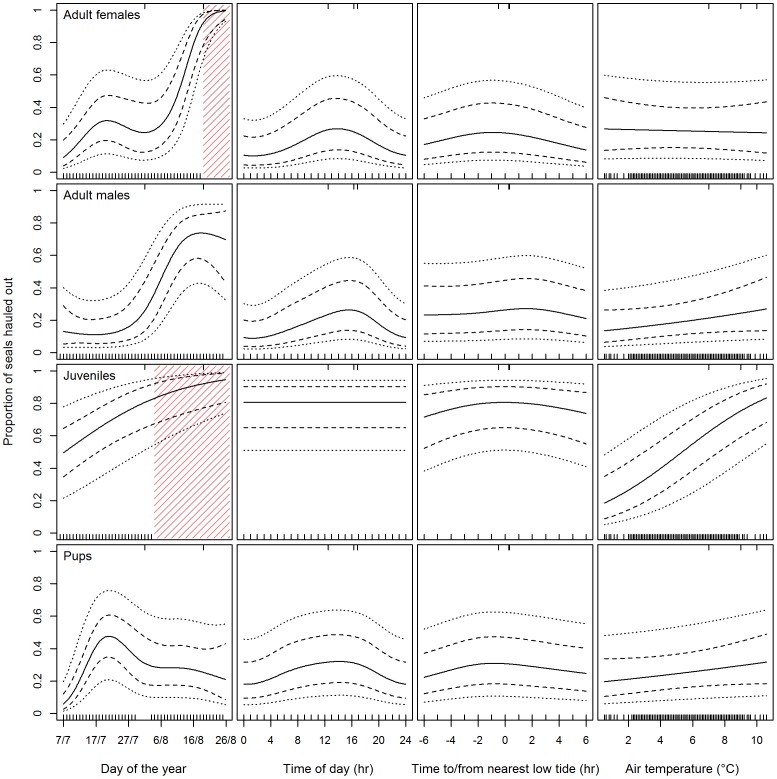
Probability model. Fitted model of the probability to haul-out for VHF-tagged seals depending on day of the year, time of day, hours to/from nearest low tide and air temperature for each of the four age/sex groups; adult females, adult males, juveniles and pups. Each panel shows the fitted predictions (solid line) using the observed values for the 01 August 2009 aerial survey for the three other predictor variables for each group. The stippled lines represent 95% confidence intervals around the mean and the outermost dotted lines show the uncertainty in the predictions for a random individual seal. The distribution of the predictor variables is shown along the bottom of each panel, and the distribution of the variables related to each aerial survey is shown along the top of each panel. Shaded areas in the panels, day of the year, for adult females and juveniles show extrapolation of the model, which are not based on raw data.

All temporal and environmental parameters (DOY, TIME, LOW TIDE and TEMP) were significant and hence they were all retained in the final model (Table 2, Figure 4). The effects of DOY for adult females and juveniles were extrapolated in the models towards the end of the study period, beyond the time period for which data was available (shaded areas in Figure 4). Estimated auto correlation between seals was 0.66 (SE - 0.026) and the between seal standard deviation on a logit scale was estimated to be 0.56 (SE - 0.102). There was no evidence for over dispersion. Estimated haul-out probabilities differed for each seal group and each survey (Table 3). The observed air temperatures during the VHF study period ranged from 0.5° to 10.5°C, encompassing the range experienced during the current survey efforts.

**Table 2:** ANOVA results with degrees of freedom (df), F value and P-values for each covariate and seal age/sex group (GROUP).

	Coefficient	df	F	P-value
Parametric	Air temperature (TEMP)	1.00	0.05	0.82
	GROUP	3.00	9.60	**<0.0001**
	TEMP * GROUP	3.00	6.73	**0.0002**
Approximate	DOY adult females	3.54	23.81	**<0.0001**
	DOY adult males	3.44	46.58	**<0.0001**
	DOY juveniles	1.00	14.58	**0.0001**
	DOY pups	3.74	16.85	**<0.0001**
	TIME adult females	2.50	14.67	**<0.0001**
	TIME adult males	2.62	15.23	**<0.0001**
	TIME juveniles	0.00	0.09	0.77
	TIME pups	2.47	9.82	**<0.0001**
	LOW TIDE adult females	2.50	6.47	**0.0007**
	LOW TIDE adult males	2.36	1.08	0.34
	LOW TIDE juveniles	2.35	3.42	**0.026**
	LOW TIDE pups	2.47	3.76	**0.017**

Day of the year (DOY), time of day (TIME) and hours to/from nearest low tide (LOW TIDE) are modelled as smooth functions. Therefore df, F values and P-values are approximations [62]. Significant values are in bold.

**Table 3:** Estimated probability for seals to haul-out for each age/sex group for each survey, as well as the proportions of each seal group within the estimated age distribution and the estimated total abundance of seals for each of the three aerial surveys based on stereoscopic images.

SurveyNo. (*t*)	Group (*j*)	Proportion of eachgroup in thepopulation (q*100)	Probability for sealsto haul-out for eachgroup (p*100)	Correction factor (CI)(1/∑(p*q))	Population estimate (CI) (*N*)
1	adult females	15	24	1.92 (1.69–3.08)	1888 (1660–3023)
	adult males	15	26		
	juveniles	46	81		
	pups	25	31		
2	adult females	15	26	2.39 (1.89–4.86)	1742 (1381–3549)
	adult males	15	19		
	juveniles	46	62		
	pups	25	27		
3	adult females	15	92	1.40 (1.28–3.41)	1812 (1656–4418)
	adult males	15	73		
	juveniles	46	90		
	pups	25	24		

**Figure 5:**
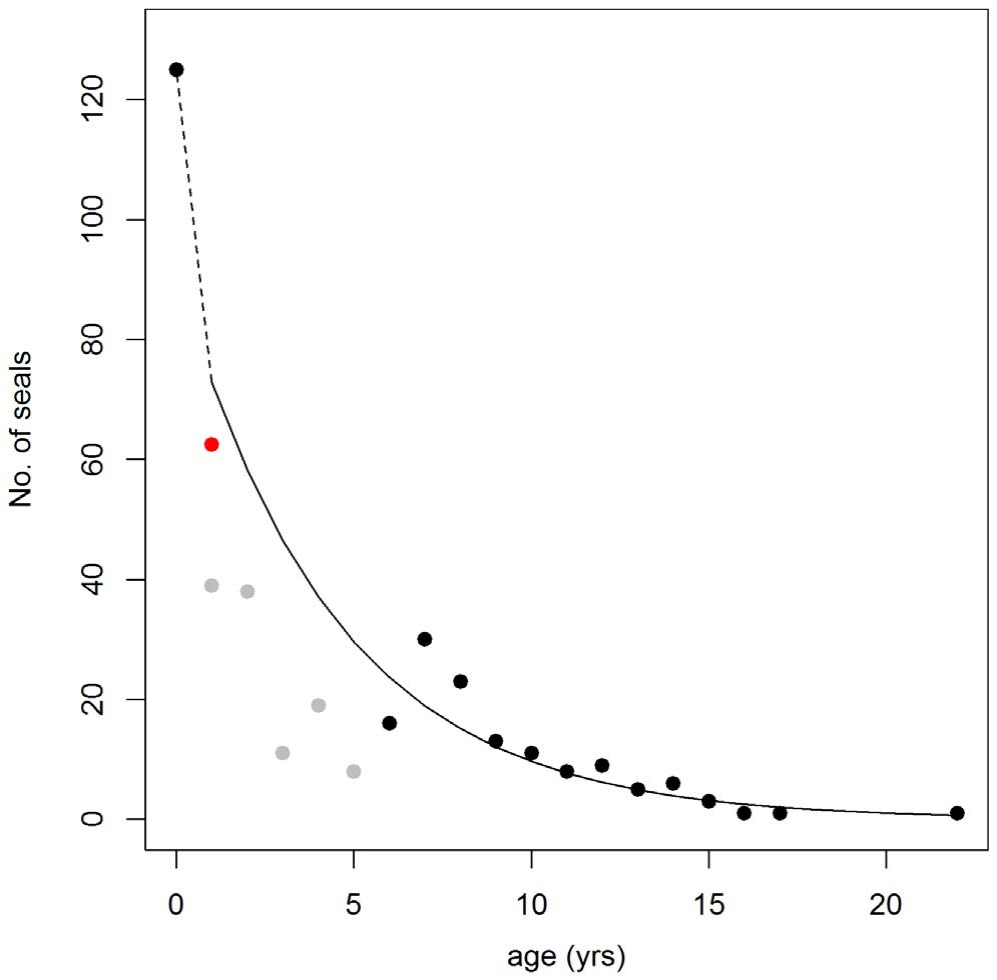
Estimated age distribution. Number of seals caught versus their age in years (black and grey circles) [5]. The solid line shows the modelled age distribution. The stippled line shows a linear extrapolation from year class 0 to year class 1. The basis for this model is age class data (6–22 yrs - black circles), and an assumed 50% pup mortality (red circle). Grey circles were excluded from the model because these age classes are assumed to be underrepresented in the capture data.

The age distribution model (R^2^ - 0.87) estimated pup mortality to be 37% while each subsequent year class had a mortality rate of 21% (Figure 5). Using this age structure and an assumed 1∶1 sex ratio for adults, the age composition of the population was estimated to be 24.7% pups, 45.8% juveniles and 14.8% for each sex among adult animals (Table 3). Varying adult sex ratios (0.77∶1 to 1∶0.77) for the estimated age distributions did not affect the population estimate (maximum difference 0.6%) and did not affect the uncertainty around the estimates (max. diff. 2%).

Combining the estimated probabilities for seals to haul-out for each age/sex group with the estimated age distribution, correction factors accounting for the proportion of animals in the water were 1.92 (CI - 1.69–3.08), 2.39 (CI - 1.89–4.86) and 1.40 (CI - 1.28–3.41) for the three surveys, respectively. These results, in combination with the aerial survey counts of the harbour seals at Prins Karls Forland produce total population estimates of 1888 (CI - 1660–3023), 1742 (CI - 1381–3549) and 1812 (CI - 1656–4418) for 01 August 2009, 01 August 2010 and 19 August 2010, respectively (Table 3).

## Discussion

The results presented herein provide the first population estimate of the Svalbard harbour seal population. Earlier abundance information for this population was based on ground-based counts spread over several days and no attempt was made to correct for individuals that were in the water at the time(s) of the surveys [1]. The haul-out probability model in the current study, combined with the modelled age distribution, gave a series of three quite similar population estimates (1742, 1812 and 1888) for the three aerial surveys conducted in two consecutive years, with the favoured estimate being 1888 (CI - 1660–3023) harbour seals residing along the west coast of Prins Karls Forland (see below).

Reader bias during counts of the survey images was considered small enough (3.3%) to be ignored in this study. The differences that did occur between the readers were derived mainly from the uncertainty in counts of disturbed individuals that had fled into the water. Viewing the images in stereo generally increased the number of seals detected on land as well as reducing misidentification of seals and surrounding rocks compared with what was found in the 2D digital images; this was the case for both readers. Individual hauled out harbour seals were very easily identified using the stereoscopic digital photographs. The 3D images enhanced the visibility of the shape of seals, increasing the contrast between them and the background rocks. However, disturbed seals that had fled into the water were more difficult to distinguish on the stereo images compared to the 2D photos. This is the reason that there was a decrease in numbers from the 2D count compared to the 3D count for the 01 August 2010 survey; this survey had the largest fraction of disturbed seals (Table 1).

The number of seals counted on the images within each survey varied. This was due to varying environmental conditions and also varying amounts of disturbance in each survey. Disturbance was highest during the 01 August 2010 survey in part because of the presence of a polar bear at Fuglehuken (Figure 2), a major haul-out site, which is normally occupied by a large number of harbour seals during favourable conditions, as seen in the other two surveys conducted in this study (Figure 2). Other possible sources of disturbance include the noise or shadow of the survey plane. The survey altitude (670 m) was sufficiently high that noise from the aircraft should not have had an impact on the behaviour of the seals. Various other studies using aircraft flying at lower altitudes (100 to 300 m), report no specific disturbance to the normal haul-out behaviour of harbour seals from the passing aircraft [11], [12], [13], [14], [15], [16], [17], [18], [19], [20], [47], [48]. Harbour seal groups do rush to the sea modestly often, without specific apparent external stimuli, in this and other populations. This behavioural trait makes repetitive surveys essential for accurate abundance determinations for populations of this species [20], [49]. This is true even for populations that are largely unaffected by human disturbance, such as the isolated population in this study, that breeds and moults in a Nature Reserve where human access is very restricted. Because of the presence of the polar bear and the high fraction of disturbed seals along the coast in the 01 August 2010 survey (for whatever combination of reasons), no attempt was made to estimate interannual differences between the surveys flown on 01 August of the two survey years. The fraction of disturbed seals during the first and third survey was small enough that the counts obtained during these survey efforts are assumed to represent precise, representative pictures of the number of hauled out harbour seals early and late in the moulting period, respectively, and hence document seasonal variation adequately.

The phenology of moulting in harbour seals shows little year to year variation within populations [30], [35]. Therefore, it can be assumed that the timing of moulting for the Svalbard population was quite similar in 2000 (VHF study) compared to 2009/2010 (aerial surveys). More seals were hauled out later in the moulting season (19 August 2010, 1295 seals) compared to earlier (01 August 981 & 730 seals in 2009 and 2010, respectively). An increase in hauled out individuals was observed particularly in the northern part of Prins Karls Forland and around the southern tip of the island (Figure 2). This suggests that these areas are mainly used by moulting seals while the more central regions around Forlandsøyane are known to be the main pupping and nursing areas [31].

Measurements of seals from the stereoscopic digital images were only possible for animals that did not move during the two seconds it takes for the camera system to construct overlapping images. For motionless animals, it was possible to determine approximate body lengths, correcting lengths in relation to the angle of the substrate the animal was hauled out on using the 3D perspective afforded by the stereo images. Assuming that the fraction of seals measured in each survey represents the real size distribution for each respective survey, no difference in length distribution was detectable between the surveys (Figure 3). This suggests that there were no age structure differences between surveyed years or seasons. However, since the Svalbard harbour seals are sexually dimorphic with regard to size [5] a potential change in age distribution could be camouflaged since immature males could be mistaken for adult females and vice versa. In addition, the resolution of the images of 4×4 cm could be too low to detect subtle changes in the length distribution of the seals.

The probability model underlying the estimated correction factor mirrored the general knowledge of harbour seal haul-out behaviour, with peak haul-out times in the afternoon, right before or during the low tide period with an increasing probability of hauling out with increasing temperature [12], [31], [32], [33], [34]. Further, juveniles were observed to increase the amount of time hauled out first, which coincides with normal behaviour during moulting documented at other locales; they were followed by adult females and then adult males, while pups gradually decreased the amount of time they spent hauled out through the study period (Figure 4) [29], [30], [31], [35]. Towards the end of the study period, a lack of data for juveniles in particular, but also adult females, led to extrapolation of the haul-out probability models (shaded areas in Figure 4). This is likely the reason for the high estimate (90%) produced for the probability of juvenile seals to haul-out during the last survey (Table 3), which is likely an overestimate for this age class. This issue is also reflected in the increased uncertainty around the population estimate for this survey (CI - 1656–4418). It is not surprising that the model provides the most accurate correction factors within the available range of the raw data.

Despite the differences in the numbers of seals counted during the three surveys, the adjusted population estimates (Table 3) are quite similar across all three surveys. This suggests that the variation in the number of seals due to the different environmental and timing factors (e.g. difference in TEMP and TIME between the first and the second survey) is being dealt with in a reasonable manner by the probability model. However, the 01 August 2009 estimate (1888 CI - 1660–3023) is thought to be the most reliable estimate, since the 01 August 2010 survey suffered from disturbance by a polar bear at an important haul-out location, and the correction factor for the 19 August 2010 survey was more uncertain due to lack of behavioural data for some age group this late in the season.

The raw data used to compute the estimated age distribution was based on 367 individual seals [5] collected over three consecutive years (1998 to 2000). During this time, the total population was assumed to be ∼1000 individuals. Therefore, the basis for the model has a high sample size compared to the total population. Modelling the age distribution, rather than using the age distribution of captured seals directly, was necessary since immature individuals were underrepresented in the dataset (grey circles in Figure 5). This underrepresentation was likely an effect of sampling taking place during the breeding period at active pupping sites, which are mainly occupied by adult individuals and pups of the year [3]. The estimated proportion of pups (0.25) produced by the model is consistent with earlier observational data from Prins Karls Forland late in the pupping period (0.24) [21]. This figure is somewhat higher than what has been found in other harbour seal studies, where the proportion of pups ranges from 0.18 to 0.20 [50]. However, the remarkable absence of older individuals in the harbour seal population in Svalbard [5] is consistent with the finding of a higher proportion of younger individuals in the total population. Further, an estimated first year mortality of 0.37 is consistent with the estimated harbour seal pup mortality (0.39) in Alaska [51]. The estimated mortality rate of 0.21 for all age classes (except pups) is also very similar to the estimated mortality rate (0.20) for all age classes in Pacific harbour seals (*P. v. richardii*) [52]. So the estimated age distribution appears to represent the age classes reasonably well. Since this age distribution assumes a stable age composition, it should not be used to explore trend analyses for this population since changes in the age composition, a potential reason for shifts in the abundance of the population, would be masked [35], [53], [54].

To assess if the assumption of a 1∶1 sex ratio for adult individuals affected the abundance estimates, age distribution models with different sex ratios, ranging from 0.77∶1 to 1∶0.77 were explored. Assuming different sex ratios made it apparent that the population estimates and their uncertainties are not sensitive to a change in the 1∶1 sex ratio assumption.

Only the coastline of Prins Karls Forland was surveyed in this study because this area was believed to be the focal haul-out area for this species in Svalbard [1], [3], [36]. However, in the last few years an increasing number of harbour seals have been observed hauling out in other areas within the Svalbard Archipelago during the summer ([55], [56], Norwegian Polar Institute Marine Mammal Sighting Database, http://mms.data.npolar.no/accounts/). Prins Karls Forland undoubtedly still represents the major breeding and moulting area for this species in this region, but due to the expanding distribution of this population it is reasonable to believe that the population of harbour seals could already be somewhat larger than the estimate(s) herein (surveys flown 2 and 3 years ago).

Accurate assessments of a total population of harbour seals in Svalbard in the future will have to be based on aerial surveys during the moulting period that encompass a broader geographic spread that at least serially encompasses the full range of the species in the region. Additionally, it is clear that replicate surveys should be flown to minimise the impact of stochastic and other acute disturbances (e.g. polar bear predation). If correction factors from this study are to be applied to future surveys, these surveys should take place within the modelled time frame and temperature range documented in this investigation, as extrapolations outside the data range markedly increase the uncertainty of the estimates. Further, an attempt should be made to derive an approximate age distribution for the population for each abundance estimate in order to detect possible shifts in age structure [35]. The approach used in this study, employing bootstrapping to derive a measure of variance for the population estimates, underestimates total variance because it does not account for variance in the estimated age distribution. But, further work is needed to access the added uncertainty.

This study has shown that an estimated 1888 (CI - 1660–3023) harbour seals were found along the west coast of Prins Karls Forland; numbers of seals were similar across surveys with largely overlapping CIs (Table 3). However, despite the fact that (1) the surveys were performed at a high altitude to avoid frightening hauled out individuals; (2) the aerial digital stereoscopic photographic surveys were performed during optimal conditions to detect the largest possible proportion of the population hauled out; (3) an attempt was made to identify age structure shifts during the moult based on length measurements of seals from stereoscopic images; (4) the results from the digital images for two of the three surveys were reliable and of high quality; and (5) the estimate was adjusted for seals in the water at the time of the surveys, following the complex haul-out behaviour of different age and sex classes of harbour seals during the moulting season and corrected for the respective proportion in the total population - it is still likely that the estimate represents a modest underestimate of the real population size, because of the expanding distribution of this population within the Svalbard Archipelago.

This small population occupies a limited spatial range [1], [3], is isolated from neighbouring harbour seal populations and demonstrates low genetic diversity [4], all of which make it vulnerable to chance events. This means that it could be at risk within the current scenario of climate change in the Arctic, particularly with respect to changing disease exposure [57]. However, for this northernmost population of harbour seals, a warming Arctic will likely have a positive impact in other regards since more suitable habitat will become available [58], and resident ice-associated seals that likely compete for food resources currently [59], [60] are expected to experience population declines in the coming decades [61]. Populations such as this one, at the edge of the species range are interesting model populations in the context of climate change.

## Supporting Information

Figure S1 **VHF data.** VHF signal records for each tagged seal. Thick lines represent periods when VHF signals were routinely received (hauled out) while thinner lines represent when the animal was likely in the water, but still in the area. The periods when at least one receiver station failed are identified with grey shading.
(TIFF)

Appendix S1 **R script.** Haul-out probability model specifications.
(R) Click here for additional data file.

Appendix S2 **R script.** Calculation of the bootstrapped population estimates.
(R) Click here for additional data file.

Appendix S3 **Test data.** Sample VHF telemetry data from Svalbard harbour seals (*Phoca vitulina*).
(TXT)
