# Supplementary figures and images for: The World’s Northernmost Harbour Seal Population–How Many Are There?
Source: PLoS One. 2013 Jul 3;8(7):e67576. doi: 10.1371/journal.pone.0067576 (PMC3701074; doi:10.1371/journal.pone.0067576)

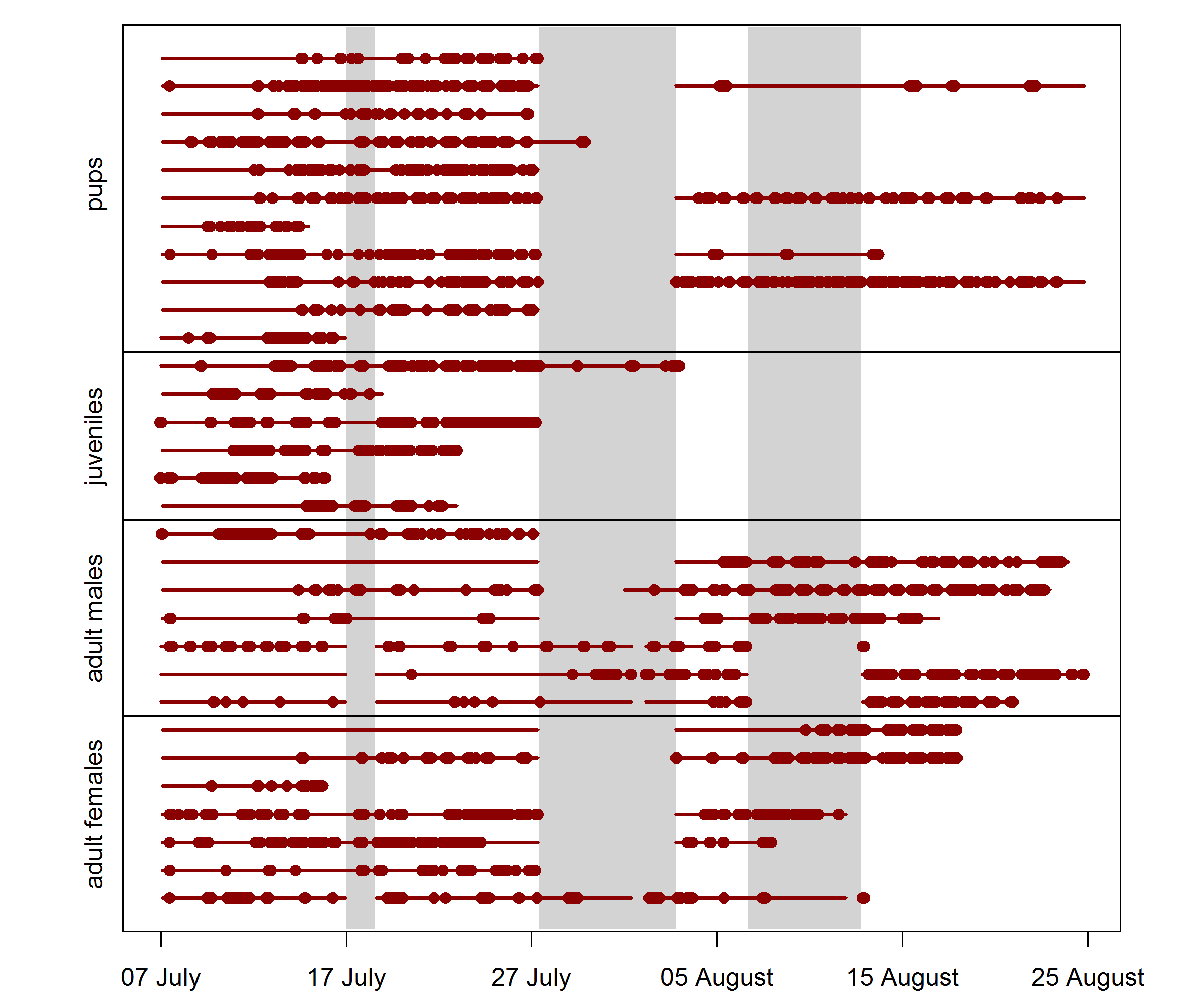

Supplement: Figure S1 — VHF data. VHF signal records for each tagged seal. Thick lines represent periods when VHF signals were routinely received (hauled out) while thinner lines represent when the animal was likely in the water, but still in the area. The periods when at least one receiver station failed are identified with grey shading. (TIFF) [file pone.0067576.s001.tiff]
